# A Novel Polarized Light Microscope for the Examination of Birefringent Crystals in Synovial Fluid

**DOI:** 10.3390/gucdd2040022

**Published:** 2024-10-22

**Authors:** John D. FitzGerald, Chesca Barrios, Tairan Liu, Ann Rosenthal, Geraldine M. McCarthy, Lillian Chen, Bijie Bai, Guangdong Ma, Aydogan Ozcan

**Affiliations:** 1 Department of Medicine, David Geffen School of Medicine, University of California, Los Angeles, CA 90095, USA; 2 Electrical and Computer Engineering Department, University of California, Los Angeles, CA 90095, USA; 3 Bioengineering Department, University of California, Los Angeles, CA 90095, USA; 4 California NanoSystems Institute (CNSI), University of California, Los Angeles, CA 90095, USA; 5 Will and Cava Ross Professor of Medicine, Medical College of Wisconsin, Milwaukee, WI 53226, USA; 6 Mater Misericordiae University Hospital and School of Medicine, University College Dublin, D04 C1P1 Dublin, Ireland

**Keywords:** crystal analysis, synovial fluid, polarized light microscopy

## Abstract

**Background:**

The gold standard for crystal arthritis diagnosis relies on the identification of either monosodium urate (MSU) or calcium pyrophosphate (CPP) crystals in synovial fluid. With the goal of enhanced crystal detection, we adapted a standard compensated polarized light microscope (CPLM) with a polarized digital camera and multi-focal depth imaging capabilities to create digital images from synovial fluid mounted on microscope slides. Using this single-shot computational polarized light microscopy (SCPLM) method, we compared rates of crystal detection and raters’ preference for image.

**Methods:**

Microscope slides from patients with either CPP, MSU, or no crystals in synovial fluid were acquired using CPLM and SCPLM methodologies. Detection rate, sensitivity, and specificity were evaluated by presenting expert crystal raters with (randomly sorted) CPLM and SCPLM digital images, from FOV above clinical samples. For each FOV and each method, each rater was asked to identify crystal suspects and their level of certainty for each crystal suspect and crystal type (MSU vs. CPP).

**Results:**

For the 283 crystal suspects evaluated, SCPLM resulted in higher crystal detection rates than did CPLM, for both CPP (51%. vs. 28%) and MSU (78% vs. 46%) crystals. Similarly, sensitivity was greater for SCPLM for CPP (0.63 vs. 0.35) and MSU (0.88 vs. 0.52) without giving up much specificity resulting in higher AUC.

**Conclusions:**

Subjective and objective measures of greater detection and higher certainty were observed for SCPLM over CPLM, particularly for CPP crystals. The digital data associated with these images can ultimately be incorporated into an automated crystal detection system that provides a quantitative report on crystal count, size, and morphology.

## 1. Introduction

The diagnosis of crystal arthritis can be established by the identification of monosodium urate (MSU) or calcium pyrophosphate (CPP) crystals from synovial fluid aspirates using a compensated polarized light microscope (CPLM). The identification of MSU crystals from a symptomatic joint or bursa is sufficient criteria for the classification of gout [1]; and the identification of CPP crystals from a joint with swelling, tenderness, or pain is sufficient criteria for the classification of disease due to CPP deposition (CPPD) [2]. Since 1961, compensated polarized light microscopy (CPLM) has been the gold standard for crystal identification in synovial fluid [3]. However, the process can be labor-intensive requiring the examination of many high-power 40× or 100× fields of view (FOV) [4]. The reproducibility of CPLM is challenging [5–8], and its sensitivity and specificity are dependent on the experience of the observer [9]. Moreover, compared to MSU crystals, CPP crystals are challenging to identify due to their smaller size (often <2 μm in length) [10] and weaker birefringence. This results in poor contrast with the magenta-colored background in CPLM. Even amongst crystal-oriented rheumatologists, CPP crystal identification errors are common (with error rates exceeding 50%) [11]. Birefringence is optimized when the crystal orientation is well aligned with the slow axis of polarization. Particularly for small, weakly birefringent crystals, misalignment affects birefringence making crystal detection more difficult.

We modified the conventional CPLM to be equipped with a polarized digital camera to image synovial fluid, aiming to enhance crystal detection [12]. This single-shot computational polarized light microscopy (SCPLM) approach benefits from a polarized complementary metal–oxide–semiconductor (CMOS) image sensor with polarized filters oriented along four diagonal axes, alleviating the restriction of crystal orientation to the slow axis of polarization. Furthermore, we added multi-focal depth imaging, generating a thicker focal plane than otherwise available with standard CPLM-based objective lenses. We have previously published single images of MSU and CPP crystals [10,12].

To evaluate the clinical utility and crystal detection rates for this new method, we generated digital Fields of View (FOV) using both SCPLM and CPLM methods from standard of care clinical samples of synovial fluid. Then, crystal experts reviewed the images, identified objects (crystal suspects) in the synovial fluid and provided their certainty that these objects were crystals as well as their determination of crystal type. Tests for the diagnostic accuracy of the images were assessed by detection rate, sensitivity, specificity, and area under the curve (AUC).

## 2. Materials and Methods

The development of clinically valid SCPLM digital images is a critical step on the path towards an automated crystal detection diagnostic instrument, but this is not meant to be a diagnostic tool when used as a stand-alone instrument. For clarity in describing our current methodology, we referred to the Standards for Reporting Diagnostic Accuracy (STARD) 2015 guidelines [13].

### 2.1. Synovial Fluid Acquisition and Slide Preparation

A convenience sample of synovial fluid samples, collected as part of routine clinical care between June 2020 and March 2021, was prepared for crystal analysis by our University Clinical Laboratory. As part of slide preparation for routine crystal analysis in our University lab, synovial fluid and contents were affixed to slides using Cytospin then Wright-stained and sealed. Once the clinical utility of the samples was exhausted, the slides were de-identified and transferred to the PI.

The lead rheumatologist screened the slides at the University Clinical Lab for the presence or absence of crystals using microscopy with white and polarized light (Olympus BX 41, Hachioji, Tokyo, Japan) and then in more detail at his research lab using Olympus BX 40 (with and without polarized and retardance filters). Areas of interest (crystal-rich regions) were noted. Slides from patients having MSU, CPP, or no crystals (5, 6, and 3 slides, respectively), as determined by the lead rheumatologist review, were then transferred to the Ozcan Engineering lab for digital imaging.

### 2.2. SCPLM/CPLM Methods

**Figure 1.**
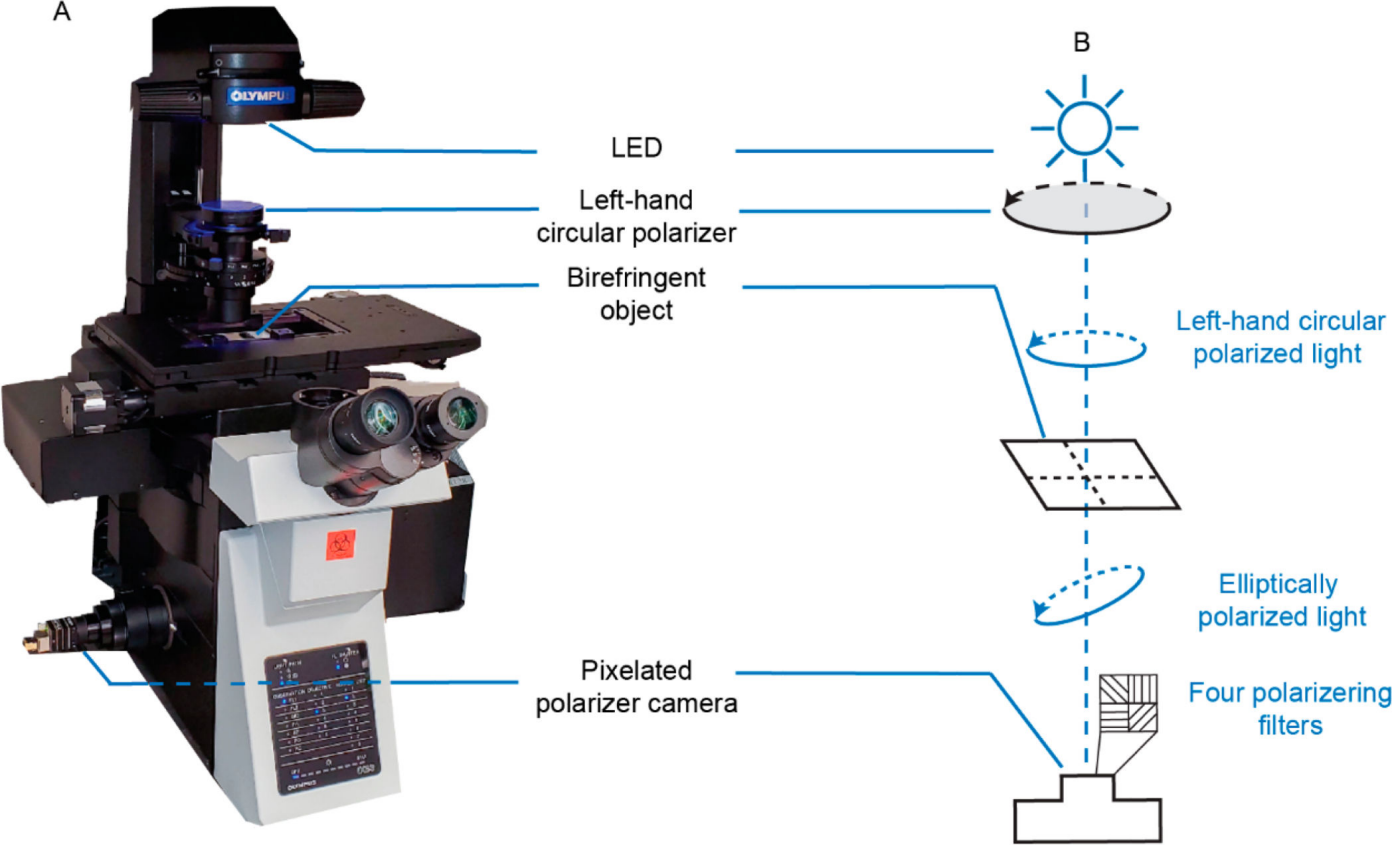
Single-shot computational polarized light microscopy (SCPLM) setup and schematic diagram. (**A**) Single-shot computational polarized light microscopy (SCPLM) setup. (**B**) Schematic diagram of the SCPLM setup.

Using the de-identified clinical slides, SPCLM and CPLM digital images from regions of interest on the slide were created concurrently in the Ozcan Research lab at UCLA (same day for each clinical slide as they became available). The setup of SCPLM is shown in Figure 1A. A conventional bright-field microscope (IX83, Olympus) is illuminated with a light-emitting diode (LED, Part# M455L3-C1, Thorlabs, Newton, NJ, USA), and modified with the following components. A left-hand circular polarizer (SKU# 88–086, Edmund Optics, Barrington, NJ, USA) is placed between the LED and the clinical sample. The elliptically polarized light is then captured by a polarization complementary metal–oxide–semiconductor (CMOS, PHX050S-PC, LUCID Vision Labs, Richmond, BC, Canada). The polarization CMOS sensor has 2448 × 2048 pixels, each with the size of 3.45 μm × 3.45 μm resulting in an image size (and FOV for the project) of 84 × 70 μm^2^ (at 100× magnification). This sensor incorporates four distinct linear polarizers oriented at 0°, 45°, 90°, and 135° for each set of 2 × 2 neighboring pixels, enabling each pixel to capture the polarization state of light along its designed orientation. A schematic illustration of this setup is shown in Figure 1B.

**Figure 2.**
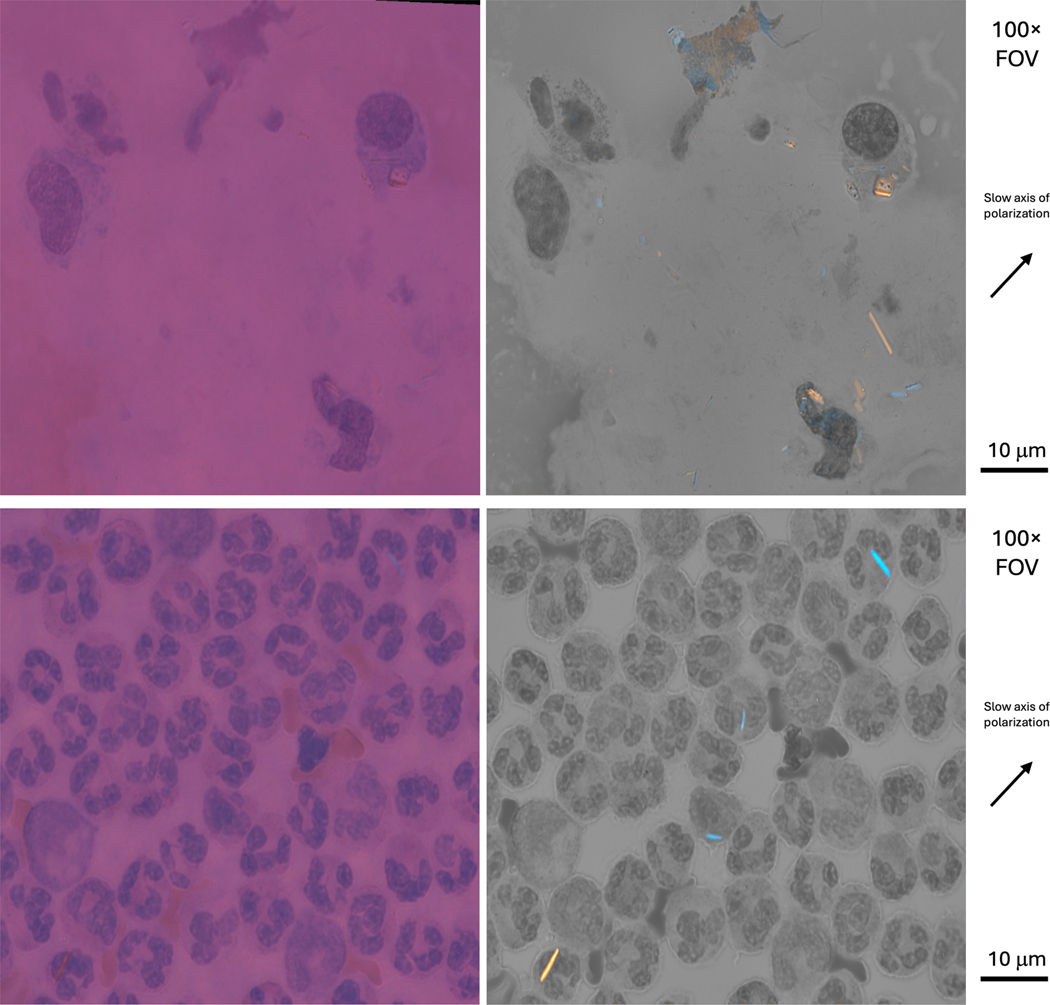
**Top** row: Side-by-side CPLM (magenta) and SCPLM (grey) comparison images (CPPD patient). **Bottom** row: Side-by-side CPLM (magenta) and SCPLM (grey) comparison images (MSU patient).

Using this SCPLM method, we reconstruct the retardance and the slow axis orientation for any birefringent object (crystal suspect) suspended in the synovial fluid, and then combine these two channels to produce a single FOV with familiar-appearing birefringent (pseudo-colored) objects. This pseudo-colored darkfield image is fused with the non-polarized light, bright FOV to create a single bright-field fused image (see Figure 2 for SCPLM examples). Finally, since the SCPLM method can capture the birefringence information in a single-shot manner at each focal depth, this method can be potentially adapted to a z-stack scanning microscope to utilize multi-focal depth, allowing for the inspection of a greater sample volume than is available with standard CPLM-based objective lenses.

For each FOV of interest, 2 CPLM images (with analyzer at orthogonal positions) were captured. CPLM captures images using a microscope (Olympus BX51) with additional compensator polarization components, including a drop-in polarizer (Model# U-POT, Olympus), a retardance plate (gout analyzer, Model# U-GAN, Olympus), and a color charge-coupled device (CCD) image sensor (Retiga-2000R, QImaging). This sensor features 1600 × 1200 pixels, each with a size of 7.4 μm × 7.4 μm, resulting in an imaging field of view of 118 × 89 μm^2^ (at 100× magnification). After bicubic interpolation and registering, the CPLM images were cropped to a uniform size of 84 × 70 μm^2^ to match the corresponding SCPLM FOV size and image area.

### 2.3. Primary Outcomes

Detection rate, sensitivity, and specificity were evaluated by presenting the 2 expert raters (described further below) with the randomly sorted FOVs of the 2 CPLM (orthogonal polarization, paired and not separated during sorting) and a single bright-field fused SCPLM for each of the 100× FOVs. Secondarily, the odds ratio (OR) for crystal detection and area under the curve (AUC) were calculated for crystal identification by SCPLM vs. CPLM.

It was not our principal objective to compare raters, but we did calculate measures of inter-rater reliability using the Ruzicka similarity coefficient and intra-class correlation coefficient (ICC).

### 2.4. Additional Secondary Outcomes

In addition to the above, rater preference (SCPLM vs. CPLM) was evaluated using side-by-side (single CPLM and single bright-field SCPLM) 100× images. Using a score sheet, raters were asked on a 1–5 scale (1 favors CPLM, 5 favors SCPLM) to indicate the favored image for detection and separately for crystal identification. See Supplementary Figure S1.

With an effort to create an SCPLM examination to approximate the CPLM clinical experience, the area of the slide encompassing the entire synovial fluid sample (containing CPPD and/or MSU crystals) was sequentially imaged using a 40×/0.7NA objective and a motorized scanning stage. These image FOVs were pasted into a single image of the entire synovial fluid sample (from 9 clinical slides, no negative controls) and uploaded to a shared Pathozoom.com website. With this image, raters could manually scan (move image in all XY directions) and zoom in/out to examine areas of interest. Raters were also provided with the original clinical materials (slides) for their independent reviews with their own CPLM equipment. The raters reported that the SCPLM scanning experience was gratifying, but that matching individual crystals between CPLM with SCPLM digital images from entire slide FOV was challenging and could not be used for comparison and therefore not further discussed.

### 2.5. Expert Rater Training

Two international crystal experts (AR, GN) were recruited as raters to detect and identify crystals. Prior to scoring each FOV digital image, we conducted ZOOM-based training sessions (roughly 30 min) where the 2 raters were provided with 10 digital image FOVs from known positive MSU and CPP samples. (These training FOVs were not part of the study dataset). During the training session, for 2 FOVs, the lead rheumatologist reviewed his CPLM and SCPLM scoring sheets with the raters. Then, each rater was asked to emulate a scoring session for an additional 8 sample FOVs (including FOVs from MSU and CPPD patient sources). For each training FOV, each rater was provided with 2 CPLM images (with analyzer at orthogonal orientation) and a single SCPLM bright-field fused image. See sample score sheet (Supplementary Figure S1). Raters were also provided with side-by-side (CPLM, SCPLM) FOV images and asked to rate their preferred image (see Figure 2).

After the training was completed, the expert clinical raters were sent study sample materials including the digital CPLM and SCPLM images on PowerPoint files (primary outcome), side-by-side digital images (secondary outcome), paper scoring sheets, and the 9 clinical slides.

### 2.6. Crystal Inclusion for Analysis

From our original 68 FOVs, 5 FOVs (all from CPPD patient sources) were excluded with more than 15 unique crystals per FOV as we could not distinguish if an unscored crystal was missed or omitted due to the 15 crystals per FOV limit. See Supplementary Figure S2 for example of a “crowded” FOV excluded from the analytic set. From the remaining 63 FOVs, any crystal suspect identified by either rater, by either CPLM or SCPLM method, was included in the analytic set.

### 2.7. Rater Evaluation of Images

In random order, raters reviewed digital images (on their computer) from 63 digital FOVs derived from 23 FOVs (CPPD clinical source), 33 FOVs (MSU clinical source), and 7 negative controls. For each CPLM or SCPLM FOV, the crystal experts were provided with a paper print out of the respective CPLM or SCPLM FOV and asked using a paper and pen method to record up to 15 crystal suspects. (See sample score sheet in Supplementary Figure S2). Raters scored the certainty that the suspect object was a crystal (range 1–5, from 1 = very uncertain, <10%; 3 = maybe, 34–67%; to 5 = very certain, >90%) and whether each crystal suspect was a CPP or MSU crystal. For 10 crystals, the identity of the crystal was unclear to the rater and was therefore labelled as “Unknown (UNK)”. For all of these UNK crystals, the crystal suspect was identified by only 1 rater (8 of 10 times with low certainty, 2 with intermediate certainty). The lead rheumatologist was not able to provide further classification for these crystals with any certainty.

This rating process generated 4 score sheets (2 raters, 2 methods) for each FOV. Crystals across the 4 scoresheets were matched so that each unique crystal became the unit of analysis.

### 2.8. Statistical Analysis

#### Accuracy Measures

Scores from both raters were divided by the max score of 5 to convert to predicted probabilities. Probabilities of 0.5 or greater (scores 3, 4, 5) were defined as a certain crystal and probabilities below 0.5 were defined as a negative rating (scores 0, 1, 2). We reported accuracy measures of detection rate, sensitivity, and specificity stratified by crystal suspect type.

Odds ratios for the detection of crystals by SCPLM over CPLM were calculated using mixed effects logistic regression to model the binary outcomes of detection and certainty based on the score assigned by raters and the predictors of both method and rater. An interaction term was additionally included in the final model if the interaction between the main effects was significant. We defined positive detection as scoring above 0, and positive certainty as scoring ≥ 3.

To evaluate inter-rater reliability, where crystal is the unit of analysis and no traditional false positive scenario exists, we calculated the Ruzicka similarity coefficient (0–1 scale where a higher number shows greater overlap) and the ICC for two-way consistency stratified by crystal suspect type [14].

Confidence intervals of 95% for AUC and inter-rater reliability measures were calculated by bootstrapping using 1000 replicates. Confidence intervals of 95% for sensitivity, specificity, and detection rate were calculated using a Clopper–Pearson binomial exact interval. All statistical analyses were performed with R version 4.4.0 [15].

### 2.9. Ethical Statement

The UCLA Institutional Review Board defined this study as non-human research.

## 3. Results

**Figure 3.**
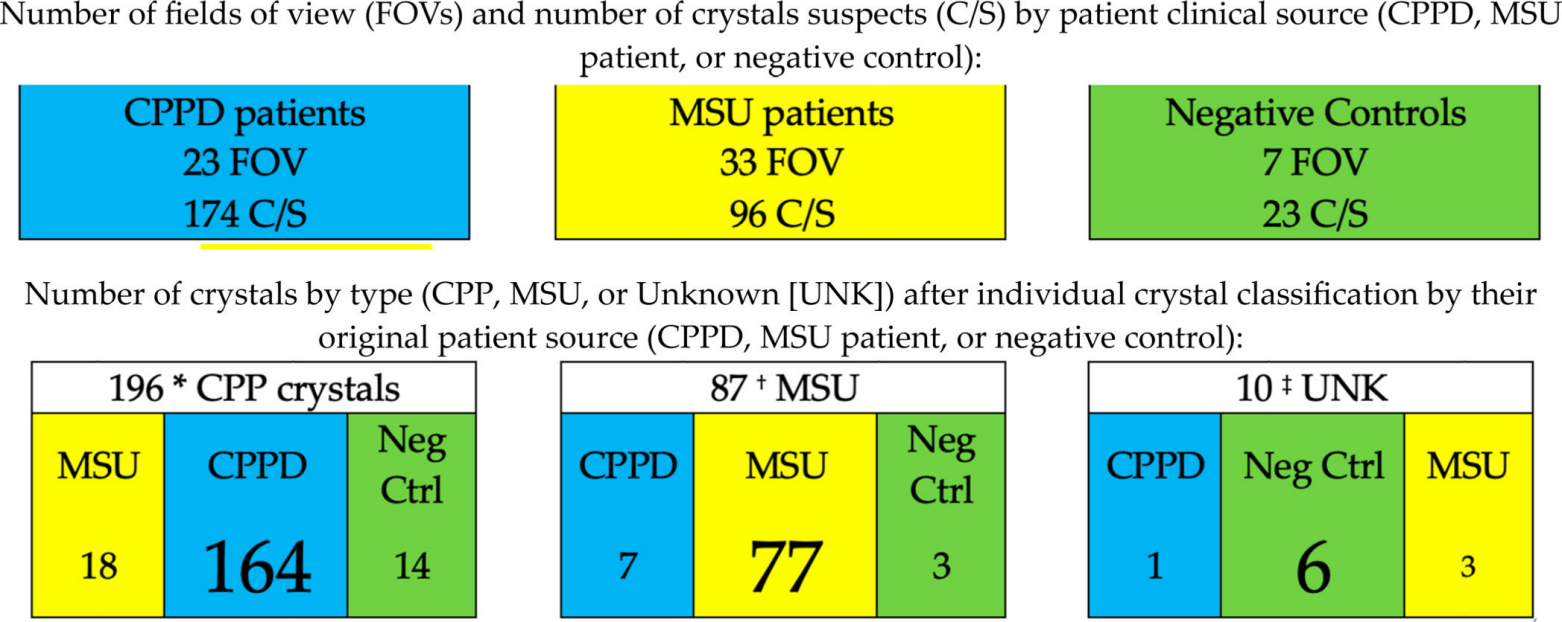
Crystal Identification Workflow and Final Crystal Specific Analytic Sample Selection Clinical Source. * 37/196 crystals with low certainty scores (1 or 2). ^†^ 10/87 crystals with low certainty scores. ^‡^ 10/10 crystals with low certainty scores and excluded from crystal-specific analyses. Legend: CPPD = calcium pyrophosphate deposition. MSU = monosodium urate. FOV = field of view. C/S = crystal suspect. UNK = unknown. Neg Ctrl = negative control.

After excluding FOVs with crowded crystals, 23 FOVs (CPPD clinical source), 33 FOVs (MSU source), and 7 FOVs (negative controls) contributed 293 crystal suspects (C/S). (Figure 3). As each crystal was the unit of analysis, raters evaluated each crystal as either MSU or CPP. For 10 crystals, raters either disagreed or designated the crystal as unknown (UNK). Not surprisingly, all 10 UNK crystals had very low rater certainty scores and most of these UNK crystals came from negative controls.

There were 18 crystals from MSU patient-sourced material that were identified as CPP by both raters, and 7 crystals from CPPD patient-sourced material that were identified as MSU by both raters. For these 25 “mismatched” crystals, the raters’ certainty scores averaged 1 point lower than the raters’ certainty ratings for crystals identified from the matching patient source (e.g., MSU crystal from MSU patient source). After crystal identification, there were 196 CPP crystals, 87 MSU crystals for crystal-specific analysis, and the 10 UNK crystals that were excluded from crystal-specific analyses.

### 3.1. Primary Outcomes: Detection Rates, Sensitivity, and Specificity

SCPLM resulted in higher crystal detection rates than did CPLM, for both CPP (51%. vs. 28%) and MSU (78% vs. 46%) crystals. Similarly, sensitivity was greater for SCPLM for CPP (0.63 vs. 0.35) and MSU (0.88 vs. 0.52) without giving up much specificity resulting in higher AUC for SCPLM for both crystals (Table 1). The odds ratio of a crystal being detected using SPCLM vs. CPLM was 4.2 with a 95% confidence interval (3.0 to 5.9, *p* < 0.001) for CPP and 30.8 (11.7 to 81.3, *p* < 0.001) for MSU crystals (OR in Table 1 and histogram shown in Supplementary Figure S3).

### 3.2. Secondary Outcomes: Rater Side-by-Side Preferences

When presented with side-by-side images, on a 5-point Likert scale where 1 = strong preference for CPLM and 5 = strong preference for SCPLM, raters strongly favored SCPLM over CPLM for both CPP and MSU types (average rating 4.85 for both) for crystal detection and (4.85 and 4.80) for crystal identification. The four slides with equivocal ratings between the methodologies were all from FOVs of negative controls and there was no preference in methodology for the negative controls (Table 2).

### 3.3. Additional Analyses: Inter-Rater Reliability

Both raters were more likely to identify more crystals using SCPLM over CPLM, particularly for MSU. Rater two identified more crystals and used higher certainty scores than did rater one, particularly for CPP crystals (Supplementary Figure S3). Inter-rater reliability between raters was moderate as measured by Ruzicka similarity (measuring overlap between distribution of ratings) and ICC (Table 1). Inter-rater agreement was higher for MSU than CPP crystals (for both CPLM and SCPLM methods).

## 4. Discussion

The validation of SCPLM digital images is the first step moving towards our goal of developing an automated crystal detection system. Given the known challenges in identifying crystals using CPLM [4], particularly for smaller, more weakly birefringent CPP crystals [10], we feel the increased SCPLM detection rates observed here are an important clinical improvement in synovial fluid crystal analysis. The digital images generated for viewing are not a final step towards a diagnostic automated crystal detection system, but the images would serve as an important quality control component for such a system.

We envision an automated crystal detection system would function similarly to an automated cell counter system. (See Supplementary Figure S3). Such a system could return a quantitative report on crystal detection beyond the current simple CPLM qualitative reports describing the presence or absence of crystals, crystal type, and crystal location (intra- or extracellular). A quantitative report could provide crystal counts, a description of morphology including the size, shape (rhomboid vs. rod), and type of crystal and the proportion of intracellular crystals detected. The clinical utility of this additional evaluation of the current flare or prognostic of future flare potential (e.g., due to crystal burden) is unknown. However, it has been reported that monoclinic (rod-shaped) crystals have more inflammatory potential than triclinic (rhomboid-shaped) CPP crystals [16]. Swan and colleagues further reported that larger crystal size and the number of CPPD crystals identified in synovial fluid correlates with the severity of the acute inflammatory attack [16].

Our SCPLM methodology has several advantages over current manual CPLM methodology that likely led to increased detection rates. These advantages include the multiple polarized filters oriented along four separate axes reducing dependency of the crystal orientation for optimal birefringence imaging. Additionally, our SCPLM technology uses multi-focal depths eliminating the need to manually pan up and down through different *Z*-axis focal planes (particularly relevant for high power 100× FOV exams with thin focal planes).

However, there are limitations to this proof-of-concept analysis. Despite enticing images and increased detection rates, the ultimate clinical utility relies upon side-by-side testing. With the polarized CMOS chip observing the data, the clinician cannot directly observe the sample (as done with CPLM) and can only observe the digital recreation of the findings. Raters were provided with two CPLM views, but neither the white light nor dark field (cross-polarized) images were provided, which a clinical rater might use during a thorough synovial fluid examination. Therefore, despite the strong findings, these data are not evidence of diagnostic superiority without further testing.

Other recent methodologies have tried to overcome limitations inherent with CPLM. Our own lab previously reported the results of lens-free polarized microscopy [17]. Lens-free methodology benefited from potential point-of-care service, large single FOV (20 mm^2^), and low-cost. With pixel super-resolution, objects 2 μm in length (covering 5 pixels) could be imaged with sufficient resolution for accurate classification. “Objects smaller than this threshold had insufficient resolution to assign a crystal type accurately” [18]. In this report, we compare lens-free and SCPLM images side-by-side. Largely attributable to superior resolution (helpful for the detection of smaller CPP crystals), development progressed with SCPLM.

Finally, in the absence of true known crystal results, (e.g., Raman scanning of each crystal—not available at our center or readily available) estimates of test performance are challenging. Recently, other authors have reported high detection rates of calcium oxalate crystals in synovial fluid (using an integrated Raman polarized light microscope) [19], and calcium oxalate monohydrate can be rod-like and positively birefringent [20]. Synthetic MSU crystals have established utility [21], but synthetic CPP crystals are challenging to fabricate (often due to odd shapes and demanding fabrication conditions) precluding use as clinically representative examples [22].

While a picture may be worth a thousand words, the potential clinical benefits of SCPLM will rely on further testing. We find the enhanced SCPLM CPP detection rates intriguing. Based on our observations, CPP crystals may be more prevalent in synovial fluid and appear more abundant than appreciated by traditional CPLM. Further studies will determine whether these observations may have implications for patient care.

## Figures and Tables

**Table 1.** Detection rates, sensitivity, specificity, and odds ratio of detecting a crystal by SCPLM compared to CPLM.

	CPP		MSU	

	N = 196		N = 87	

Estimates (95% confidence interval)	CPLM	SCPLM	CPLM	SCPLM
Detection rate	0.28 (0.24, 0.33)	0.51 (0.46, 0.56)	0.46 (0.39, 0.54)	0.78 (0.71, 0.84)
Sensitivity	0.35 (0.30, 0.40)	0.63 (0.58, 0.68)	0.52 (0.44, 0.61)	0.88 (0.82, 0.93)
Specificity	0.79 (0.68, 0.88)	0.76 (0.65, 0.85)	0.95 (0.76, 1.0)	0.75 (0.51, 0.92)

Odds ratio (OR) of crystal detected by SCPLM vs. CPLM (controlling for rater differences)	OR 4.2, *p* < 0.001		OR 30.8, *p* < 0.001	
AUC	0.57 (0.51, 0.62)	0.73 (0.68, 0.79)	0.76 (0.71, 0.8)	0.85 (0.77, 0.94)

Inter-rater reliability Ruzicka similarity	0.37 (0.28, 0.47)	0.43 (0.37, 0.49)	0.66 (0.54, 0.78)	0.65 (0.56, 0.73)
ICC (2-way mixed effects, consistency)	0.47 (0.36, 0.58)	0.37 (0.24, 0.49)	0.69 (0.56, 0.79)	(0.11, 0.49)

**Table 2.** Rater preference for SCPLM vs. CPLM by clinical source.

	CPPD	MSU	Negative Control

FOV rated (N)	N = 28 ^1^	N = 32 ^2^	N = 7
Mean Rating for detection (range)	4.85 (3‒5)	4.85 (3‒5)	3.4 (3‒5)
Mean Rating crystal identification (range)	4.85 (3‒5)	4.80 (3‒5)	3.1 (2‒5)

Legend: 5-point Likert scale where 1 = strong preference for CPLM and 5 = strong preference for SCPLM. 1 Includes 5 FOVs with 15 or more crystals that were excluded from the analysis of crystals; 2 1 missing rater response.

## Data Availability

The original contributions presented in the study are included in the article/Supplementary Material, further inquiries can be directed to the corresponding author.
